# Identification of an infectious bronchitis coronavirus strain exhibiting a classical genotype but altered antigenicity, pathogenicity, and innate immunity profile

**DOI:** 10.1038/srep37725

**Published:** 2016-11-23

**Authors:** Shu-Yi Lin, Yao-Tsun Li, You-Ting Chen, Ting-Chih Chen, Che-Ming J. Hu, Hui-Wen Chen

**Affiliations:** 1 Department of Veterinary Medicine, National Taiwan University, Taipei, Taiwan; 2 Institute of Biomedical Sciences, Academia Sinica, Taipei, Taiwan; 3 Research Center for Nanotechnology and Infectious Diseases, Taipei, Taiwan

## Abstract

Avian coronavirus infectious bronchitis virus (IBV) poses economic threat to the poultry industry worldwide. Pathogenic IBV 3575/08 was isolated from broilers vaccinated with the attenuated viral vaccine derived from a Taiwan strain 2575/98. In this study, extensive investigations were conducted on the genome sequences, antigenicity, pathogenicity, and host immune responses of several IBV strains in specific-pathogen-free chickens. Sequence analyses revealed that 3575/08 and 2575/98 shared high homology in their structural genes, but not in non-structural accessory proteins such as 3a, 3b and 5b. Despite a high degree of homology in their spike protein genes, cross neutralization test showed low cross protection between 3575/08 and 2575/98, suggesting distinct antigenicity for the two strains. Animal challenge experiments exhibited strong respiratory and renal pathogenicity for 3575/08. In addition, early and prolonged viral shedding and rapid viral dissemination were observed. Immune gene expression profiling by PCR array showed chickens infected with 3575/08 had delayed expression of a subset of early innate immune genes, whereas chickens infected with the wild-type or attenuated-type 2575/08 revealed quick gene induction and efficient virus control. In summary, this study reveals a new IBV strain, which harbors a known local genotype but displays remarkably altered antigenicity, pathogenicity and host defenses.

Avian coronavirus infectious bronchitis virus (IBV) is a member of *Coronaviridae* family, and the virus is responsible for infectious bronchitis, an acute, highly contagious chicken viral disease. Respiratory signs such as coughing, sneezing, and nasal discharge are the primary symptoms associated with IBV infections, and some of the strains also display kidney and oviduct tropisms, causing urogenital symptoms^1^,^2^,^3^. The disease poses economic threat to the poultry industry worldwide. More than 50 serotypes of IBV have been documented since the first isolation of virus in Massachusetts (Mass), US in the 1930 s^2^. The most commonly detected genotype of IBV in the US is Ark-DPI, accounting for 23% to 65% of total isolation per year^4^. While most strains circulating in the US cause respiratory symptoms, nephropathogenic QX-like strains are dominant in Europe^2^. In the Far East, nephropathogenic LX4, LDL as well as proventricular-type QX-like strains have been reported frequently^2^. Presently, vaccination is adopted to manage the disease, and Mass type vaccine H120 is the most widely used vaccine formulation^2^,^5^. Due to frequent strain variations, however, traditional Mass type vaccines can be ineffective against infections by different IBV variants^2^,^5^,^6^. Effective disease management thus demands better elucidation of strain variations.

In Taiwan, viral surveillance from 2005 to 2006 in poultry slaughterhouses showed the IBV flock prevalence was 17% and the chicken prevalence ranged from 1.8 to 3.7%^7^. Most of the IBV isolates in Taiwan are nephropathogenic strains^8^,^9^. Also, IBVs in Taiwan are distinct from other genotypes in the world and can be divided into two subgroups, Taiwan Group I (TW-I) and Taiwan Group II (TW-II)^10^,^11^. As TW-I strains begin to emerge in neighboring areas in recent years^12^,^13^,^14^, there is a rising urgency to thoroughly investigate the TW IBV strains.

IBV virion has a linear, positive sense and single-stranded genomic RNA. Its 27.6 kb genome contains at least ten open reading frames (ORFs), 5′-1a-1ab-S-3a-3b-E-M-5a-5b-N-3′, encoding four structural proteins and several non-structural proteins (nsp)^3^. The four structural proteins include spike glycoprotein (S), envelope protein (E), membrane glycoprotein (M), and nucleocapsid protein (N). Of the different structural proteins, the S protein, which comprises of S1 and S2 subunits, is responsible for host cell attachment and virus-cell membrane fusion and is the primary antigen target for virus neutralization. Currently, genetic grouping for IBV is based primarily on the S1 gene^1^,^2^,^3^. Among the genes that encode for non-structural proteins, the ORF 1a and 1ab encode two polyproteins, which are further cleaved into 15 nsps. These and other non-structural accessory proteins 3a, 3b, 5a and 5b are associated with RNA replication, virulence, and host-pathogen interactions of IBV^3^. However, the functions of these nsps remain largely unknown and warrant further studies.

Host innate antiviral immunity plays a critical role in viral clearance. In general, toll like receptor (TLR) families, retinoic acid-inducible gene I (RIG-I) and melanoma differentiation-associated gene 5 (MDA5, encoded by IFIH1 gene) are intracellular pattern-recognition receptors (PRRs) that function as viral infection detectors and all contribute to type I interferon (IFN) production^15^. As RIG-I is absent in chickens, IFIH1 is a functional compensate^16^. Avian TLRs are different from their mammalian counterparts, lacking TLR9 but possessing TLR1LA, poultry-specific TLR15, and a TLR9 homologue TLR21^17^. TLR3 and TLR7 are known to be involved in RNA virus recognition, and its stimulation leads to activation of IRF3 and IRF7 (IFN-regulatory factor 3 and 7). These factors then signal IFN transcription to trigger IFN-stimulated genes (ISGs) through JAK-STAT pathway to express various antiviral proteins^15^,^17^,^18^. A global gene expression profiling of chicken kidney has revealed that 103 immune and inflammatory-related genes are involved in the host defense response during the first week of IBV infection^19^. The TLR3/7 and MDA5 signaling pathways and innate immune cytokine were induced after IBV infection in chicken tracheas at 3–8 days post IBV M41 infection^20^. In addition, STAT1 expression has also been found to be significantly elevated in response to IBV infection *in ovo*^21^. However, there is a lack of understanding of early antiviral immune responses induced by various IBV strains, particularly responses triggered by strains with close genetic relationship but distinct pathogenicity.

To control IBV in Taiwan, an embryo-attenuated strain (2575 AT) derived from IBV TW-I strain 2575/98 wild type (2575 WT) has been developed as a vaccine^8^. These two strains were previously investigated to have close antigenicity^8^ and limited sequence changes^22^. However, a novel TW-I strain 3575/08 emerged from a broiler chicken farm where the 2575 AT vaccine was applied. IBV 3575/08 has been observed to cause high morbidity and mortality in affected broilers. In the present study, we compared the viral genome sequences of IBV 3575/08 and 2575/98 and investigated the infectivity and host immunity induced by 3575/08, 2575 WT, and 2575 AT infections. To this end, sequence analysis, neutralization test, experimental infection, and immune-related gene profiling were performed. The results show that IBV 3575/08, which possesses heterologous ORF 3a, 3b, and 5b sequences to 2575/98, caused a more deadly infection with early and prolonged viral shedding, stronger clinical and pathological manifestation, and delayed innate immune responses as compared to 2575 WT and 2575 AT.

## Results

### 3575/08 and 2575 WT share sequence homology except in 3a, 3b, and 5b genes

**Figure 1:**
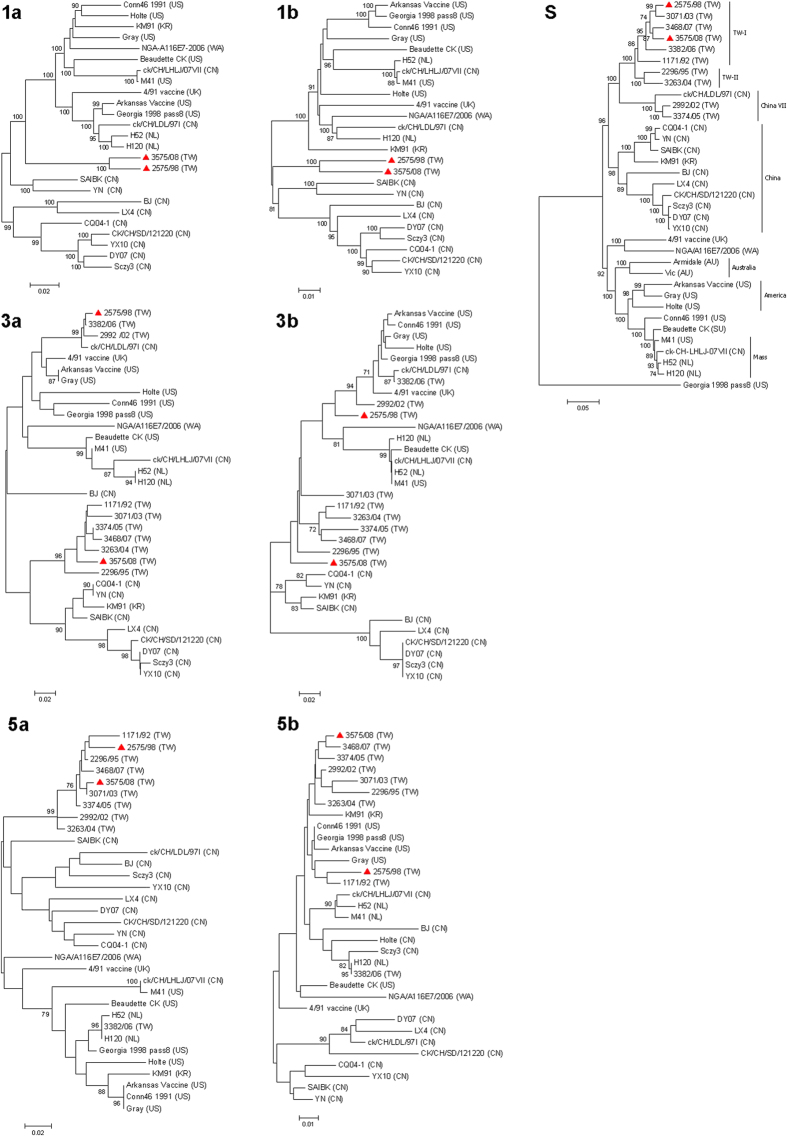
Phylogenetic analyses of 1a, 1b, S, 3a, 3b, 5a and 5b protein genes. The phylogenetic trees were constructed using the MEGA software version 4 by the neighbor-joining method (bootstrapping for 1,000 replicates with its value >70%). The 3575/08 and 2575/98 strains analyzed in this study are indicated with red triangles. (AU: Australia, CN: China, NL: Netherlands, TW: Taiwan, UK: United Kingdom, US: United States, WA: Western Africa)

The complete genome of IBV 3575/08 was sequenced in this study, and the sequences were submitted to GenBank with the accession number KX266757. Excluding the 5′ and 3′ untranslated regions the entire genome size is 26,704 nucleotides. The genome comprises ten ORFs in the order of 5′-1a-1ab-S-3a-3b-E-M-5a-5b-N-3′. No significant protein truncation was observed. To evaluate the nucleotide sequence identities among 3575/08 and other reference IBV strains, sequence alignment and phylogenetic analyses on each gene segment were performed (Fig. 1 and Supplementary Fig. S1). Comparisons of the available full-length genome showed that the sequence identities between 3575/08 and other IBV strains ranged from 85.0% (with strain BJ) to 94.9% (with strain 2575/98). Overall, IBV 3575/08 showed close genetic grouping with 2575/98 and other Taiwan local strains in 1a, 1b, S, E, M, 5a and N genes. The sequence similarities of these protein genes between 3575/08 and 2575/98 were 95.6% (1a), 94.2% (1b), 95.6% (S), 93.3% (E), 92.4% (M), 93.4% (5a), and 96.3% (N) for the respective genes. Further comparison of the two spike protein subunits between 3575/08 and 2575/98 showed sequence similarities of 97.1% and 94.4% in the S1 and S2 subunits, respectively. Based on the S gene, 3575/08 was clearly defined as a TW-I strain. The sequence identities of S gene between 3575/08 and other analyzed strains ranged from 67.6% (with strain Georgia 1998 pass8) to 96.4% (with strain 3468/07). While 3575/08 was closely related to 2575/98 in four structural protein genes, their homology in ORF 3a (86.1%), 3b (82.1%), and 5b (95.5%) were relatively low (4.5% difference is considered significant for the 5b protein since its variation among IBV strains is small). According to the phylogenic tree (Fig. 1), IBV 3575/08 and 2575 WT are grouped in different clusters with respect to their 3a, 3b and 5b genes.

### Identification of IBV 3575/08 as a unique serotype

To investigate the antigenic serotype of the newly emerged TW-I strain 3575/08, cross (two-way) neutralization tests against three major serotypes in Taiwan—TW-I, TW-II and Mass type— were performed in specific-pathogen-free (SPF) chicken embryos. The neutralizing titers and the r-values were then analyzed (Table 1 and Supplementary Table S1). With the r-value of homologous virus-serum set as 100, the r-value directly correlates with the relatedness of the strains. The results showed that all the r-values obtained in this study were below 30, indicating all the tested strains belonged to different serotypes. Particularly, TW-I 3575/08 conferred poor cross neutralization not only to heterogeneous Mass type H120 (r-value = 1.1) or TW-II 2296/95 (r-value = 3.4) but also to homologous TW-I 2575/98 (r-value = 3.3), supporting IBV 3575/08 as a unique serotype.

### IBV 3575/08 showed enhanced pathogenicity as compared to 2575 WT and 2575 AT in chickens

**Figure 2:**
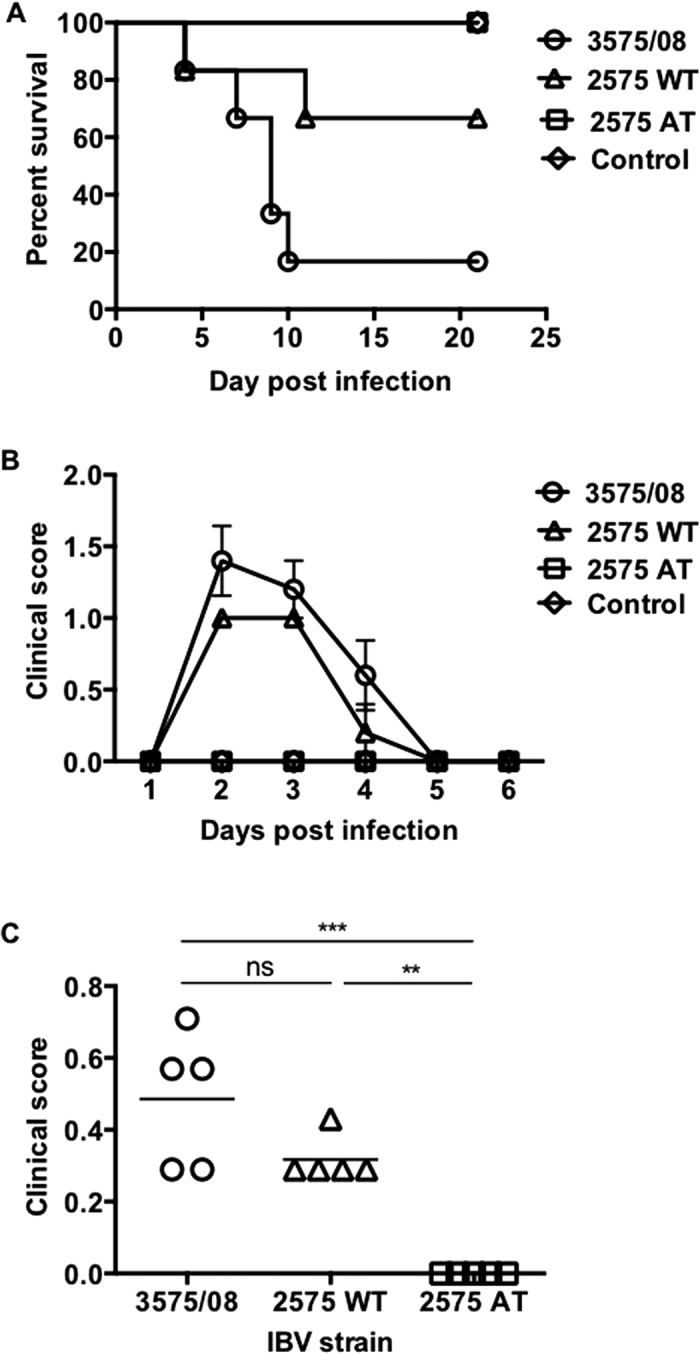
Percentage survival and clinical score of IBV infected chickens. (**A**) Survival rate of 1-day-old chicks after intranasal infection with IBV 3575/08, 2575 WT, 2575 AT. Chickens receiving PBS were served as control. (n = 6 per group) (**B**) Following intranasal infections in 2-week-old chickens, the daily averages of observed clinical scores within 6 days were recorded. Data was presented as means ± s.e.m. (n = 5 per group) (**C**) Mean clinical scores of each group during the observation period are indicated (n = 5 per group). See Methods for detailed clinical score interpretation. ns: non-significant. **P < 0.01. ***P < 0.001.

To investigate the pathogenicity of IBV 3575/08, 2575/98 WT and 2575 AT, SPF chickens were intranasally challenged with 10^6^ EID_50_ of the three strains and monitored for survival and clinical signs on a daily basis. In 1-day-old chicks during a 21-day observation period, IBV 3575/08 infection resulted in the lowest survival rate (16.7%, 1/6) as compared to 2575 WT (66.7%, 4/6), 2575 AT (100%, 6/6), and PBS control (100%, 6/6) (Fig. 2A). The earliest death from 3575/08 or 2575 WT-infected group was found on day 4 post infection (dpi).

In addition, clinicopathological assessment for the three IBV strains was performed. Following intranasal challenge in 2-week-old chickens with 10^6^ EID_50_ of the different IBV strains, the clinical scores (Fig. 2B,C) were recorded and analyzed (n = 5 per group, see Methods for detailed clinical score interpretation). It was observed that beginning at 2 dpi, chickens infected with 2575 WT exhibited moderate tracheal rales (score = 1), persisting through 3 dpi (score = 1) and resolved at 4 dpi (score = 0.2) (Fig. 2B). IBV 3575/08-infected chickens showed similar but more severe clinical signs at 2 dpi (score = 1.4) and 3 dpi (score = 1.2). At 4 dpi, more than half of 3575/08-infected chickens still suffered from mild respiratory symptoms (score = 0.6), and clinical signs receded after 5 dpi. In contrast, IBV 2575 AT-infected and PBS control groups did not show any clinical manifestations throughout the observation period. Collectively, over a 7-day observation period, the averaged clinical scores of IBV 3575/08-, 2575 WT-, 2575 AT-infected chickens were 0.49, 0.32, and 0, respectively (Fig. 2C). Overall, IBV 3575/08 infection caused stronger clinical manifestations in chickens than 2575 WT and 2575 AT.

**Figure 3:**
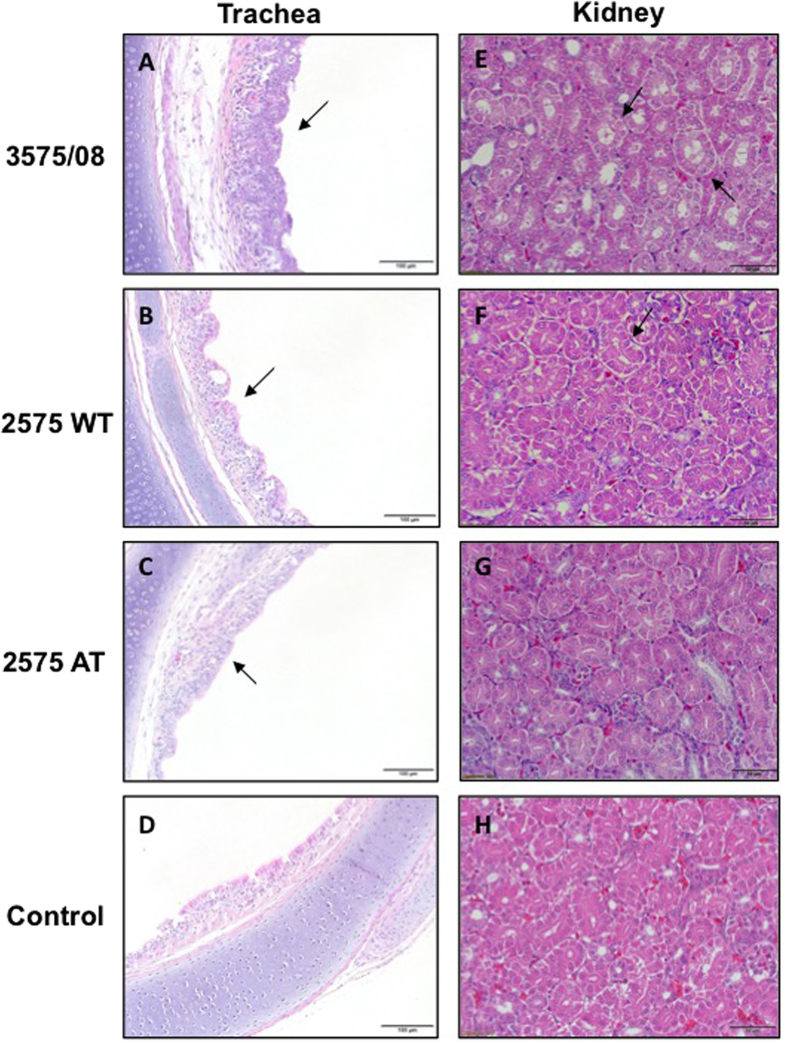
Pathological examination of trachea and kidney from IBV infected chickens. The pathological lesions of experimentally infected chickens were evaluated at 7 dpi, and representative pictures were shown. Panels D and H refer to control tissues. IBV 3575/08 infected chickens presented pathological lesions such as desquamation of ciliated cells, infiltration of heterophils, and lymphocytes and epithelial hyperplasia in the trachea (**A**, arrowed). The lesions in 2575 WT (**B**) and 2575 AT (**C**) groups were similar to the 3575/08 group but milder. For IBV 3575/08-infected chicken kidney, renal tubule dilation, necrotic tubular cells, and urate in the tubular cavity were observed (**E**, arrowed). Vesicular degeneration was found in renal tubular epithelial cells from the 2575 WT group (**F**, arrowed). For the 2575 AT group, the renal cells were normal as the control group (**G**).

The pathological lesions of experimentally infected chickens were further evaluated at 7 dpi, and representative pictures are shown in Fig. 3. In the trachea epithelium, normal goblet cells and ciliated cells were seen in the PBS control group (Fig. 3D). However, the IBV 3575/08 infected chickens presented pathological lesions such as desquamation of ciliated cells, infiltration of heterophils, and lymphocytes and epithelial hyperplasia in the trachea (Fig. 3A, arrowed). Lesions in the 2575 WT (Fig. 3B) and 2575 AT (Fig. 3C) groups were similar but less severe as compared to the 3575/08 group. Upon observing the kidneys, the control group showed normal renal tubular epithelial cell with homogeneous plasma and distinctive renal tubular cavities (Fig. 3H). In the kidneys of IBV 3575/08-infected chickens, however, renal tubule dilation, necrotic tubular cells, and urate in the tubular cavity were observed (Fig. 3E, arrowed). Vesicular degeneration was also observed in the renal tubular epithelial cells from the 2575 WT group (Fig. 3F, arrowed). On the other hand, the 2575 AT group showed normal kidney patterns similar to the control group (Fig. 3G). The pathological findings indicate that both IBV 3575/08 and 2575 WT are respiratory and nephropathogenic strains. IBV 3575/08 showed higher virulence as it inflicted more severe trachea and renal tissue damages in chickens than the 2575 WT strain. No virulence in the kidney was observed for IBV 2575 AT.

### More rapid and prolonged viral dissemination of IBV 3575/08 in chickens

To further investigate the viral dissemination of IBV 3575/08, 2575 WT, and 2575 AT in chickens, swabs and tissues were harvested from virus-infected chickens (10^6^ EID_50_, intranasal) at various time points. Viral detection was carried out by reverse transcription polymerase chain reaction (RT-PCR). As indicated in Table 2, 100% of the throat swabs from all groups were detected positive at 1, 2, 3, 7, 14 dpi. At 21 dpi, both the 2575 WT and 2575 AT groups showed a decreased positive rate at 66.7% (2/3), whereas a 100% (3/3) positive rate remained for the 3575/08 group. More importantly, for the cloacal swabs, virus was detected from 3575/08-infected chickens as early as 1 dpi (60%, 3/5), whereas the earliest time points for 2575 WT and 2575 AT were 2 dpi (40%, 2/5) and 3 dpi (20%, 1/5), respectively. There was no detectable level of viruses in any of the swab samples from the PBS control.

To examine viral distributions, the lungs and kidneys, two major organs associated with IBV tropism, were harvested at 3, 7, 21 dpi. As indicated in Table 3, whereas all of the infected chickens from the 3575/08 or 2575 WT groups were detected positive in the lungs at 3 and 7 dpi, only 2/3 and 1/2 chickens were detected positive in the 2575 AT group. As for the kidneys, virus was detected from the 3575/08-infected chickens as early as 3 dpi, whereas no virus was detected from the 2575 WT group until 7 dpi. In addition, IBV 3575/08 was detected in the trachea, proventriculus, bursa, and oviduct from experimentally infected chickens at 7 dpi (data not shown). In the 2575 AT group, no animal was detected positive in the kidneys across the indicated time points. By 21 dpi, virus was cleared from the lungs and kidneys in every group. These results show that IBV 3575/08 causes more rapid and prolonged dissemination and shedding in chickens as compared to the other two strains. The results also highlight the nephropathogenic nature of 3575/08 and 2575 WT in chickens.

### 3575/08 infection elicited a similar level of antibody titer as 2575 WT in chickens

**Figure 4:**
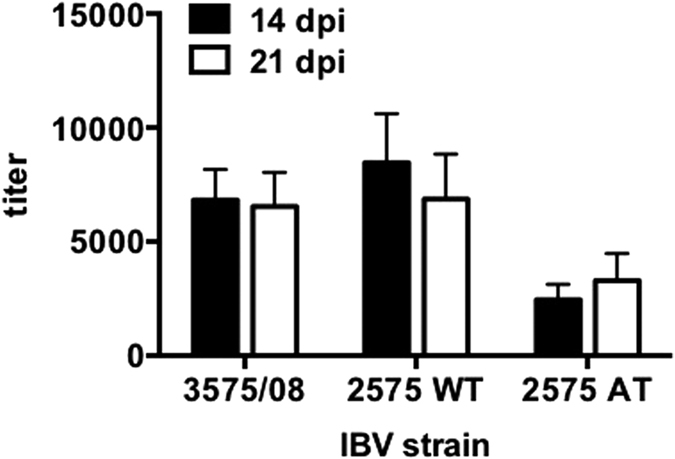
Serum antibodies of IBV infected chickens. Chicken serum was collected at 14 and 21 dpi, respectively. ELISA was performed to evaluate the antibody level against IBV. Bars represent means ± s.e.m. (n = 5 per group).

To examine the antibody response elicited by IBV infection in chickens, sera were collected from the 3575/08-, 2575 WT-, and 2575 AT-infected groups at 14 and 21 dpi. Antibody titers were analyzed by an IBV antigen coated-ELISA, and 3575/08 and 2575 WT infections elicited similar mean titer levels of 6,547 and 6,870 at 21 dpi respectively (Fig. 4). The antibody titer elicited by 2575 AT infection was lower (3,294) at 21 dpi. Similar titer results were obtained from the different infection samples at 14 dpi.

### Antiviral immune response was delayed in IBV 3575/08-infected chickens

**Figure 5:**
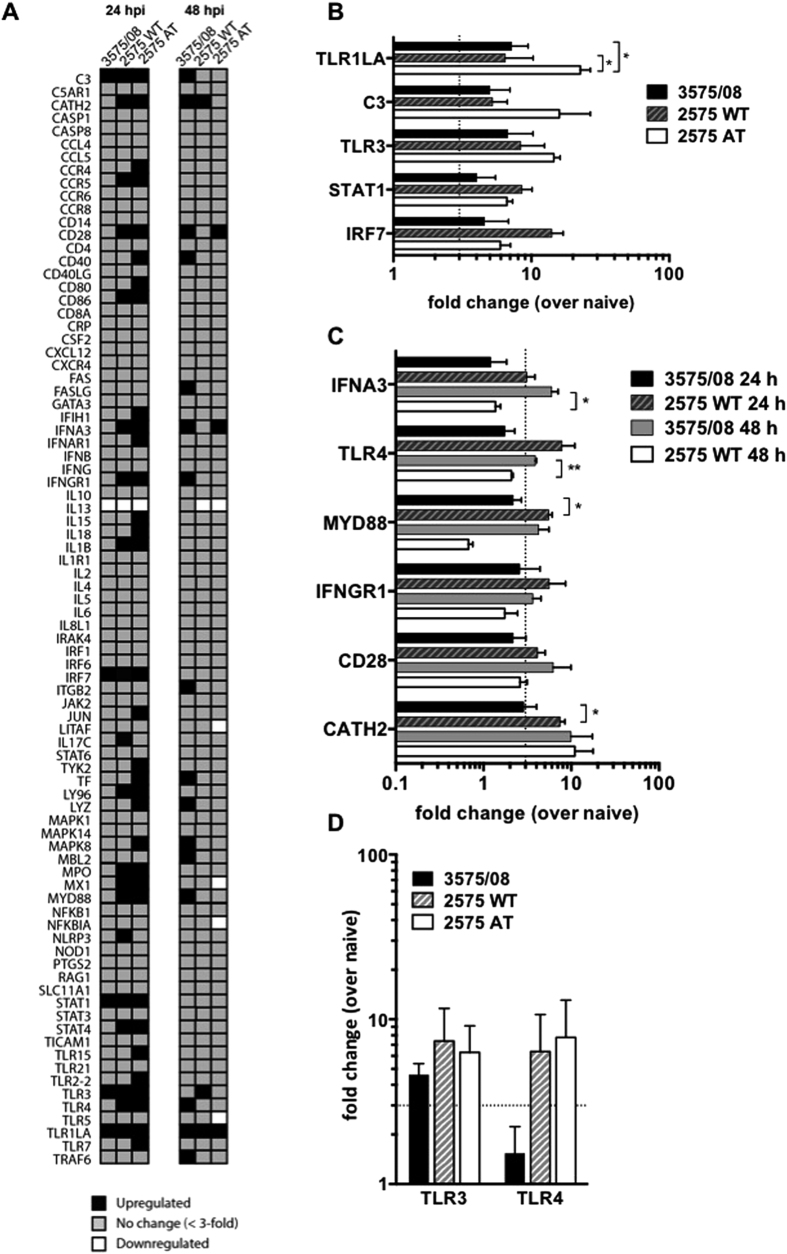
Comparison of immune related genes in IBV infected chickens. (**A**) Profiles of immune related gene expression in the lungs after infection by different strains as evaluated by RT^2^ Profiler Chicken Innate & Adaptive Immune Responses PCR Array at 24 and 48 hpi. Gene expression of transcripts with a difference of more than 3-fold over naive was illustrated (black, upregulated; gray, no change; white, downregulated). (**B**) Gene induction in the lungs of IBV 3575/08, 2575 WT, and 2575 AT-infected chickens at 24 hpi was expressed as fold-increase over naive. (**C**) Gene induction in the lungs of IBV 3575/08 and 2575 WT-infected chickens was expressed as fold-increase over naive at 24 or 48 hpi. (**D**) Expression level of TLR3 and TLR4 at 24 hpi in was validated by real-time RT-PCR. Fold changes were compared by ANOVA followed by Dunnett multiple comparisons tests. Dotted lines denote a 3-fold change over naive chickens. Bars represent means ± s.e.m. (n = 3 per group). *P < 0.05, **P < 0.01.

Next, to dissect the antiviral gene induction in chickens in response to IBV 3575/08, 2575 WT and 2575 AT infections, we examined a panel of immune response gene expression in the lung at 24 and 48 hours after the IBV infections using a quantitative PCR array kit. The fold changes in the expression of 84 genes from infected chickens were normalized to that of naive chickens (Fig. 5A, also see Supplementary Table S3 for statistical data and Supplementary Fig. S2 for the Venn diagram). At 24 hours post infection (hpi), 20 (23.8%) and 33 (39.3%) of the genes were induced by more than 3-fold over the naive control in the 2575 WT- and 2575 AT-infected chickens respectively, whereas only 5 (6%) of the 84 genes were upregulated in the 3575/08-infected chickens. At 48 hpi, 16 (19%), 3 (3.6%), and 3 (3.6%) genes were induced by more than 3-fold over the naive group in the 3575/08-, 2575 WT-, and 2575 AT-infected chicken, respectively. Overall, a robust immune-related gene expression was rapidly induced in the 2575 WT- and 2575 AT-infected chickens at 24 hpi, and a 90% overlap was observed among the upregulated genes between the two groups. These results identified the critical components in early host defense against IBV 2575 WT and 2575 AT. At the same time point, 3575/08-infected chickens showed heightened expression of only 5 innate immunity genes (TLR1LA, TLR3, IRF7, STAT1, and C3) (Fig. 5B). 6 other genes upregulated by 2575 WT and AT at 24 hpi, including TLR4, MyD88, IFNA3, IFNGR1, CD28, and CATH2, showed delayed stimulation following the 3575/08 infection (Fig. 5C). In addition, 10 other genes that were not enhanced in either the 2575 WT or the 2575 AT group were found to be upregulated in the 3575/08 group at 48 hpi. The finding suggests that IBV 3575/08 may have evaded the initial virus sensing and delayed the signaling pathways for early antiviral defense. In 2575 WT-infected chickens, the genes upregulated at 48 hpi (TLR1LA, TLR3, and CATH2) overlapped with those at 24 hpi. Similarly, the genes upregulated by 2575 AT at 48 hpi (TLR1LA, IFNA3, and CD28) also overlapped with those observed at the earlier time point. In addition, quantification of TLR3 and TLR4 expression levels by regular real-time RT-PCR was carried out to validate the results from the PCR array. The expression patterns of these two genes were consistent between the real-time PCR and the PCR array analysis, showing activation of TLR3 but not TLR4 level at 24 hpi in the 3575/08 group (Fig. 5D).

## Discussion

The spread of TW type IBV in Taiwan has been attributed to poor protection by commercial Mass type vaccines^23^. An effective TW type vaccine strain 2575 AT has been previously developed through serial passages in SPF embryonated eggs to control IBV infection in Taiwan. However, a new IBV strain 3575/08 emerged from 2575 AT-vaccinated broiler chickens and caused high morbidity and mortality. To characterize the new strain 3575/08, comparative analyses on viral genotype, serotype and pathogenicity were conducted.

Pairwise sequence comparison and phylogenetic analysis revealed that 3575/08 and 2575/98 share high homology in most of the structural protein genes, including the S1 gene that are commonly applied for IBV strain genotyping. Therefore, IBV 3575/08 was grouped in the TW-I genotype with 2575/98 and other strains. It has been reported that the serotype of IBV is associated with the hypervariable region (HVR) 1 in the N-terminus (residues 45–114) of the S1 gene^24^, which overlaps with one of the antigenic sites located in the spike protein of IBV^25^. Despite high sequence similarities between 3575/08 and 2575/98 were found in the spike protein gene (95.6%), S1 subunit gene (97.1%) and HVR 1 (94.8%), a distinct serotype of 3575/08 was clearly established in this study through a cross neutralization test. Similar findings were previously reported in UK showing that IBV strains of three distinct serotypes possess as little as 2% of amino acid variations in region 19–122 and 251–347 of the S1 subunit^26^. The variations led to failure of cross-protection^27^, supporting that small sequence differences can result in a serotype change. In addition, it has been reported that small sequence changes in the S2 subunit may alter interactions with the S1 subunit, which could in turn affect the conformation of the S1 subunit and serotype-specific epitopes^28^. A previous study on murine hepatitis virus (MHV), a group II coronavirus, also showed that a single amino acid substitution in region 1109–1116 of S2 subunit can confer resistance to neutralization by S1 subunit-specific monoclonal antibody^29^. Therefore, the gene variations in the S2 subunit (5.6%) between 3575/08 and 2575/98 may have a significant contribution to the serotype difference. Future studies are warranted to further examine the determinants of antigenicity.

In addition to the new serotype, we also found that IBV 3575/08 exhibited higher mortality and more profound clinical signs as compared to 2575 WT and 2575 AT through experimental challenges in chickens. Following intranasal infection, IBV 3575/08 had a very short latent period. The viral shedding from cloaca was detected as early as 24 hpi and lasted for 21 days, and a rapid viral dissemination to renal tissues (3 dpi) was observed. As it has been documented that spike proteins play a major role in determining viruses’ cell tropism and pathogenicity^30^, the increased IBV 3575/08 virulence is a notable finding given the high degree of homology between the spike protein genes between IBV 3575/08 and 2575 WT.

A previous study has shown that different IBV strains can cause differing IFN levels at various organs and time points in chickens^31^. We hypothesized that a different innate immune response may account for the altered pathogenicity of IBV 3575/08. Therefore, the early immune-related gene expression in the lung following 3575/08, 2575 WT and 2575 AT infections in chickens was analyzed by PCR arrays. In contrast to the 20 and 33 genes activated in the 2575 WT- and 2575 AT-infected chickens, only a small subset of the genes was triggered in the 3575/08-infected chickens at 24 hpi, and a delayed activation was observed at 48 hpi. This result suggests that IBV 3575/08 has the potential to evade the initial virus sensing and suppress the first wave of host defense, leading to enhanced pathogenicity observed in this study. Many coronaviruses have the ability to evade detection by the host’s immune system and disrupt the host immune response through multiple mechanisms^32^,^33^,^34^. It has been reported that small accessary proteins 3a and 3b of IBV can delay the onset of IFN response by interfering with the PRR sensing without disrupting the signaling pathways^35^. Previous studies have also demonstrated that 3a and 3b accessory proteins of IBV are not essential for replication but can modulate the IFN-β response at the transcriptional and translational level^36^,^37^. In addition, 5b accessory protein of IBV has been found to shut off host immune response and limit interferon production^38^. It has been shown that a natural recombinant strain containing partial nsp regions from a low pathogenic H120 strain in the frame of a nephropathogenic strain possesses attenuated pathogenicity, which suggests strong associations between nsps and IBV virulence^39^. Studies on other coronaviruses such as MHV and severe acute respiratory syndrome coronavirus (SARS-CoV) have also shown that nsps are associated with viral replication and evasion of host innate immune response via viral RNA modification^40^,^41^, host mRNA degradation, and IFN antagonist competition^42^. Therefore, given the low sequence homology in the accessory proteins 3a, 3b, and 5b between IBV 3575/08 and 2575 WT, we conjecture that these accessary proteins contribute to the blockade of PRR expressions, delayed immune response, and enhanced virulence of IBV 3575/08 in the present study. Future studies are warranted to further dissect the roles of IBV 3575/08’s nsps and accessary proteins in modulating host’s antiviral defenses. An *in vitro* infection model with detailed functional analysis of nsps would shed clearer insights on the host-virus interactions.

Also of note is that the number of PRR genes activated at the early time point correlated with IBV virulence in the study. For instance, IBV 2575 AT infection activated multiple PRR genes, including IFIH1, TLR1LA, TLR2-2, TLR3, TLR4, TLR7, and TLR15. In addition to the two primary sensors for IBV, TLR3 and TLR7^43^, other PRR molecules have been studied for their roles in coronavirus infections. In previous studies, SARS-CoV and MHV infections have been shown to trigger IFIH1 expression for MDA5 signaling^44^,^45^. It has also been reported that TLR4 expression can be induced by SARS-CoV infection^46^. The robust expression of PRR molecules triggered by IBV 2575 AT sheds light on the molecular mechanism behind the strain’s attenuated virulence and pathogenicity. In contrast, the lower number of stimulated PRR genes triggered by IBV 3575/08 and 2575 WT serve to explain these viruses’ increased virulence.

The present study may also offer insight on IBV disease management and vaccine design. Firstly, identification of a new pathogenic IBV strain with a unique serotype and strong host tropism suggests a rapid viral mutation rate in chicken hosts. This observation raises the need for regular vaccine update to keep up with the mutating viral strains. As the antigenicity or pathogenicity shift may result from genetic changes outside of structural protein genes, antigen derived from a single epitope (eg. spike protein) may not be sufficient in eliciting cross-protection against different serotypes. Multi-epitope vaccine formulations should be considered for better disease management. Secondly, the present study elucidates several key innate immune genes and TLR pathways (eg. TLR3, TLR4, MYD88, IRF7, IFNA3, etc.) responsible for early restriction of IBV infection. When designing an inactivated or subunit vaccine, immunomodulatory adjuvants may be tailored to promote an infection-like immune stimulation as observed in the study. Triggering innate immune gene expressions and TLR pathways in a virus-like manner may facilitate a robust adaptive immune response against IBV infection.

In summary, this study compares the sequence, antigenicity, pathogenicity, and immune host defenses among IBV strains. The strain 3575/08, which possesses heterologous ORF 3a, 3b, and 5b sequences, exhibits a distinct serotype and strong clinicopathological manifestation. This virus can also evade the initial virus sensing and delay several early innate immune genes, collectively leading to enhanced pathogenicity in a chicken host.

## Methods

### Ethics statement

Experiments involving chickens were conducted at National Taiwan University, under an approved Institutional Animal Care and Use Committee (IACUC) protocol (no. NTU-103-EL-3). All animal experiments were carried out in accordance with the approved guidelines.

### Virus

IBV strain 2575/98 was isolated in Changhua, Taiwan in 1998^8^, and designated 2575 wild-type (2575 WT) throughout this study. Embryo passage 74 of 2575/98 was obtained as previously described^8^, and designated 2575 attenuated-type (2575 AT) throughout this study. IBV strain 2296/95 was isolated in Taoyuan, Taiwan in 1995^8^. Mass type strain H120 was purchased from Abic Biological Lab (Beit Shemesh, Israel). IBV strain 3575/08 was isolated from a broiler farm in Yunlin, Taiwan in October 2008. In that affected farm, death was first observed in 25-day-old broilers with typical IB disease symptoms. The morbidity was higher than 15%, and the mortality was approximately 7.5%. All IBVs used in this study were propagated in 10-day-old SPF embryonated eggs (Animal Health Research Institute, Tamsui, Taiwan) via an allantoic route as previously described^47^. All the used viruses were ensured free of other avian respiratory pathogens, ie. avian influenza virus and Newcastle diseases virus contamination by RT-PCR^48^. IBV viral titer (egg infective doses 50%, EID_50_) was evaluated in 10-day-old SPF eggs and calculated by Reed-Muench method^49^.

### Viral genome sequencing and sequence analysis

Viral RNA was extracted from virus-infected allantoic fluid using a viral nucleic acid extraction kit (Geneaid Biotech Ltd., Taipei, Taiwan) following the manufacturer’s protocol. Reverse transcription was carried out with M-MLV reverse transcriptase (Invitrogen, Carlsbad, CA), and genome segments were amplified by PCR using primers listed in Supplementary Table S3. The PCR reaction mixture contains 0.5 μl of Phire hot start II DNA polymerase (Thermo, Carlsbad, CA), 5 μl of 5X buffer, 0.5 μl of 50 μM forward primer and reverse primer (Tri-I Biotech, Taipei, Taiwan), 3 μl of 2.5 mM dNTPs (GeneTeks BioScience, Taipei, Taiwan), 5 μl of cDNA and 10.5 μl of nuclease-free water. For PCR, the initial denaturation was at 95 °C for 3 min, followed by 35 cycles of denaturation at 95 °C for 1 min, annealing at 55 °C for 30 s, and extension at 72 °C for 2 min. The final extension was at 72 °C for 10 min. All the amplified PCR products were analyzed in 1% agarose gels electrophoresis. DNA sequencing was conducted by a commercial service (Tri-I Biotech). Each nucleotide was determined from at least three identical results generated from separate PCR products. Nucleotide sequences were assembled using DNAStar software (DNAStar, Madison, WI). Sequences of the reference IBV strains were retrieved from GenBank with the accession numbers listed in Supplementary Table S4. Multiple sequence alignments and sequence identity were analyzed with the Clustal W method available in BioEdit 7.2.5 software. Phylogenetic trees were constructed with the neighbor-joining method using MEGA software version 4^50^, and the bootstrap values were determined from 1,000 replicates of the original data.

### Antisera production and cross neutralization test

Antisera against 3575/08, 2575/98 and 2296/95 were prepared in 3-week-old SPF chickens as previously described^24^. The antiserum against Mass-type H120 was purchased from Charles River Laboratories (North Franklin, CT). Cross neutralization test was performed with the protocol previously described^24^. Briefly, four-fold diluted sera were incubated with the same volume of 100 EID_50_ homologous or heterologous virus at room temperature for an hour, respectively. The virus-serum mixtures were then inoculated into the allantoic cavity of 10-day-old SPF embryonated eggs. Seven days after inoculation, the eggs were opened and examined for typical lesions caused by IBV infection (embryo dwarfing or malformation). The neutralizing titer of each serum against homologous or heterologous virus was determined by the last serum dilution that protected 50% of the embryo and calculated by Reed-Muench method^49^. In addition, the cross neutralization r-values between strains were calculated by the method described by Archetti and Horsfall^51^. Antigenic (serotype) difference between two given strains was defined as follows, when r: 70–100%, same serotype; r: 33–70%, different subtype (minor); r: 11–32%, different subtype (major); r; 0–10%, different serotype^52^.

### IBV infections in SPF chickens

For survival evaluation after IBV infection, 1-day-old SPF chicks were randomly divided into three groups and intranasally challenged with 10^6^ EID_50_ of IBV 3575/08, 2575 WT and 2575 AT, respectively (n = 6 per group). Another six chickens received PBS as naïve controls. For the clinicopathological assessment, 2-week-old SPF chickens were used. Following intranasal challenge with 10^6^ EID_50_ of IBV 3575/08, 2575 WT and 2575 AT, chickens were monitored on a daily basis. The clinical scores of IBV were interpreted according to the methods described by Avellaneda *et al*.^53^. The clinical signs were evaluated as: 0 = no clinical signs; 1 = slight lacrimation, slight shaking, watering feces or tracheal rales; 2 = lacrimation, presence of nasal exudate, depression, water feces, apparent sneezing or cough; 3 = same as 2 but stronger with severe watery feces; 4 = death. Throat and cloacal swabs were collected at 1, 2, 3, 7, 14, 21 dpi for virus detection. In addition, lungs and kidneys were also harvested from euthanized chickens at 3, 7, 21 dpi for virus detection and histopathological examination. Sera were collected at 14 and 21 dpi to test the IBV antibody titer. For PCR array analysis, three chickens at 24 and 48 hpi from each challenged group were euthanized to harvest lungs. Four chickens received PBS as naive controls. For all chicken experiments, each virus-challenged group of chickens was reared in separated rooms, and utensils were separately handled to avoid any cross contamination.

### Viral detection by RT-PCR

The viral nucleic acid extraction kit (Geneaid) was used to extract the viral RNA from swabs samples and tissue homogenates. One step RT-PCR was performed in a total reaction volume of 25 μl containing 0.5 μl Phire hot start II DNA polymerase (Thermo), 5 μl 5X buffer, M-MLV RT (200 U/μl) (Invitrogen), 1 μl RNaseOUT (40 U/μl) (Invitrogen), 0.5 μl 50 μM forward primer (5′-GGTAGYGGYGTTCCTGATAA-3′) and reverse primer (5′-TCATCTTGTCRTCACCAAAA-3′) (Tri-I Biotech) for a 618 bp fragment of IBV N gene detection, 3 μL 2.5 mM dNTPs (GeneTeks BioScience), 2.5 μl RNA, and 10.5 μl nuclease-free water. RT-PCR was performed at 40 °C for 30 min, 98 °C for 3 min, followed by 35 cycles of denaturation at 98 °C for 30 s, annealing at 58 °C for 30 s, and extension at 72 °C for 30 s, and the final extension was performed at 72 °C for 3 min. PCR products were then analyzed on 2% agarose gels.

### ELISA

A flat-bottomed microplate (Nunc, Denmark) was coated overnight with purified IBV antigen (100 ng/well) with coating buffer (15 mM Na_2_CO_3_ and 35 mM NaHCO_3_, pH 9.6). The wells were washed three times with PBST (0.1% (v/v) Tween 80 in PBS) and blocked with 5% (w/v) skim milk (BD Difco, Sparks, MD) at room temperature for 1 h. After blocking, serial diluted chicken serum was added to each well (100 μl/well) and incubated at room temperature for 1 h. Following three washes, 100 μl of 1:2,000 diluted peroxidase-conjugated affinipure rabbit anti-chicken IgG (H + L) (Jackson ImmunoResearch, West Grove, PA) was dispensed into each well and incubated at room temperature for 1 h. After three additional washes, the wells were then incubated with 100 μl of SureBlue Reserve TMB Microwell Peroxidase Substrate (KPL, Gaithersburg, MD) for 10 min in the dark. The reaction was stopped by the addition of 100 μl of TMB stop solution (KPL). The optical density (OD) at 450 nm was read using an automated plate reader (Thermo).

### Histopathological examination

Chicken tracheas, lungs, and kidneys were collected and fixed in 10% formalin (Sigma, MO) at room temperature for at least 48 h. Fixed tissues were embedded in paraffin wax, and cut into 6 μm sections. Sections were then routinely processed and stained with hematoxylin and eosin (H&E). Microscopic lesions resulted from IBV infection were examined by light microscopy.

### PCR array

To analyze the innate and adaptive immune responses in chicken after infection, RT^2^ Profiler Chicken Innate & Adaptive Immune Responses PCR Array (PAGG-052ZG; SABiosciences) was used following the manufacturer’s protocol. Total RNA in chicken lung was extracted using TriSolution Reagent Plus (GMbiolab, Taipei, Taiwan) following the recommended protocol for tissue samples and quantitated using spectrometry. To avoid the contaminations of genomic DNA, 1 μg total RNA was treated with 1 U DNase I (Thermo) for 30 min at 37 °C, then 1 μl EDTA was added and incubated 10 min at 60 °C to inactivate the DNase. cDNA was synthesized using the RT^2^ First Strand kit (SABiosciences), mixed with 2× RT^2^ qPCR Master Mix (SABiosciences), and then loaded onto the 384-well PCR array plate for amplification on the LightCycler 480 PCR system (Roche). The steps of the cycling program were 95 °C for 10 min followed by 40 cycles of 95 °C for 15 s and 60 °C for 1 min. Data were analyzed using the online software provided by SABiosciences (http://pcrdataanalysis.sabiosciences.com/pcr/arrayanalysis.php).

### Real-time RT-PCR

Total RNA in chicken lung was extracted as described above. Real-time RT-PCR was performed with QuantiNova Reverse Transcription kit (Qiagen, Alameda, CA) and QuantiNova SYBR Green PCR kit (Qiagen) using previously described primers that target the chicken TLR3, TLR4, and 28S^54^. All reactions were set up in duplicate on the iQ5 real-time PCR system (Bio-rad). The steps of the cycling steps were 95 °C for 2 min followed by 40 cycles of 95 °C for 5 s and 60 °C for 10 s. Obtained cycle threshold (Ct) values were normalized to 28 S. The relative expression of TLR3 and TLR4 (fold change of naive control) was determined using the 2^−ΔΔCt^ method^55^.

### Statistical analysis

Data were analyzed by unpaired t tests or ANOVA followed by Dunnett’s multiple comparison tests using GraphPad Prism (GraphPad Software, San Diego, CA). The *p* values smaller than 0.05 were considered significant.

## Additional Information

**How to cite this article**: Lin, S.-Y. *et al*. Identification of an infectious bronchitis coronavirus strain exhibiting a classical genotype but altered antigenicity, pathogenicity, and innate immunity profile. *Sci. Rep.* **6**, 37725; doi: 10.1038/srep37725 (2016).

**Publisher's note:** Springer Nature remains neutral with regard to jurisdictional claims in published maps and institutional affiliations.

## Supplementary Material

supplementary Tables and Figures

## Figures and Tables

**Table 1:** R-value of cross neutralization test with TW-I strains (3575/08, 2575/98), TW-II strain (2296/95) and Mass type (H120).

IBV strain	3575/08 (TW-I)	2575/98 (TW-I)	2296/95 (TW-II)	H120 (Mass)
3575/08 (TW-I)	**100**			
2575/98 (TW-I)	3.3	**100**		
2296/95 (TW-II)	3.4	12	**100**	
H120 (Mass)	1.1	2.6	12.8	**100**

**Table 2:** Rate of IBV detection by RT-PCR in chicken throat and cloacal swabs after infections.

IBV strain	Throat swab % IBV positive					
	1 d	2 d	3 d	7 d	14 d	21 d
3575/08	100% (5/5)	100% (5/5)	100% (5/5)	100% (5/5)	100% (3/3)	100% (3/3)
2575 WT	100% (5/5)	100% (5/5)	100% (5/5)	100% (5/5)	100% (3/3)	66.7% (2/3)
2575 AT	100% (5/5)	100% (5/5)	100% (5/5)	100% (5/5)	100% (3/3)	66.7% (2/3)
Cloacal swab % IBV positive						
3575/08	60% (3/5)	60% (3/5)	100% (5/5)	100% (5/5)	100% (3/3)	100% (3/3)
2575 WT	0% (0/5)	40% (2/5)	100% (5/5)	100% (5/5)	100% (3/3)	100% (3/3)
2575 AT	0% (0/5)	0% (0/5)	20% (1/5)	80% (4/5)	100% (3/3)	100% (3/3)

**Table 3:** IBV detection by RT-PCR in chicken lungs and kidneys at 3, 7, and 21 day post infection.

IBV strain	Lung			Kidney		
	3 d	7 d	21 d	3 d	7 d	21 d
3575/08	3/3	2/2	0/3	1/3	2/2	0/3
2575 WT	3/3	2/2	0/3	0/3	2/2	0/3
2575 AT	2/3	1/2	0/3	0/3	0/2	0/3

## References

[b1] Cook J. K., Jackwood M. & Jones R. C. The long view: 40 years of infectious bronchitis research. Avian Pathol. 41, 239–250 (2012). 22702451 10.1080/03079457.2012.680432

[b2] Jackwood M. W. Review of infectious bronchitis virus around the world. Avian Dis. 56, 634–641 (2012). 23397833 10.1637/10227-043012-Review.1

[b3] Cavanagh D. Coronavirus avian infectious bronchitis virus. Vet. Res. 38, 281–297 (2007). 17296157 10.1051/vetres:2006055

[b4] Sjaak de Wit J. J., Cook J. K. & van der Heijden H. M. Infectious bronchitis virus variants: a review of the history, current situation and control measures. Avian Pathol. 40, 223–235 (2011). 21711181 10.1080/03079457.2011.566260 PMC7154306

[b5] Jackwood M. W. . Data from 11 years of molecular typing infectious bronchitis virus field isolates. Avian Dis. 49, 614–618 (2005). 16405010 10.1637/7389-052905R.1

[b6] Feng J. . Virulent avian infectious bronchitis virus, People’s Republic of China. Emerg. Infect. Dis. 18, 1994–2001 (2012). 23171741 10.3201/eid1812.120552 PMC3557894

[b7] Chen H. W., Huang Y. P. & Wang C. H. Identification of intertypic recombinant infectious bronchitis viruses from slaughtered chickens. Poult. Sci. 89, 439–446 (2010). 20181858 10.3382/ps.2009-00322 PMC7107050

[b8] Huang Y. P. & Wang C. H. Development of attenuated vaccines from Taiwanese infectious bronchitis virus strains. Vaccine 24, 785–791 (2006). 16239054 10.1016/j.vaccine.2005.08.081 PMC7115542

[b9] Lin K. Y., Wang H. C. & Wang C. H. Protective effect of vaccination in chicks with local infectious bronchitis viruses against field virus challenge. J. Microbiol. Immunol. Infect. 38, 25–30 (2005). 15692623

[b10] Wang C. H. & Tsai C. T. Genetic grouping for the isolates of avian infectious bronchitis virus in Taiwan. Arch. Virol. 141, 1677–1688 (1996). 8893790 10.1007/BF01718291 PMC7086916

[b11] Huang Y. P., Lee H. C., Cheng M. C. & Wang C. H. S1 and N gene analysis of avian infectious bronchitis viruses in Taiwan. Avian Dis. 48, 581–589 (2004). 15529980 10.1637/7186-033004R

[b12] Xu G. . Characterization and analysis of an infectious bronchitis virus strain isolated from southern China in 2013. Virol. J. 13, 40, doi: 10.1186/s12985-016-0497-3 (2016). 26955947 PMC4784446

[b13] Xu Q. . Emergence of novel nephropathogenic infectious bronchitis viruses currently circulating in Chinese chicken flocks. Avian Pathol. 45, 54–65 (2016). 26551660 10.1080/03079457.2015.1118435

[b14] Ma H. . Genetic diversity of avian infectious bronchitis coronavirus in recent years in China. Avian Dis. 56, 15–28 (2012). 22545524 10.1637/9804-052011-Reg.1

[b15] Barber G. N. Innate immune DNA sensing pathways: STING, AIMII and the regulation of interferon production and inflammatory responses. Curr. Opin. Immunol. 23, 10–20 (2011). 21239155 10.1016/j.coi.2010.12.015 PMC3881186

[b16] Barber M. R., Aldridge J. R. Jr., Webster R. G. & Magor K. E. Association of RIG-I with innate immunity of ducks to influenza. Proc. Natl. Acad. Sci. USA 107, 5913–5918 (2010). 20308570 10.1073/pnas.1001755107 PMC2851864

[b17] Chen S., Cheng A. & Wang M. Innate sensing of viruses by pattern recognition receptors in birds. Vet. Res. 44, 82, doi: 10.1186/1297-9716-44-82 (2013). 24016341 PMC3848724

[b18] Alexopoulou L., Holt A. C., Medzhitov R. & Flavell R. A. Recognition of double-stranded RNA and activation of NF-κB by Toll-like receptor 3. Nature 413, 732–738 (2001). 11607032 10.1038/35099560

[b19] Cong F. . Transcriptome analysis of chicken kidney tissues following coronavirus avian infectious bronchitis virus infection. BMC Genomics 14, 743, doi: 10.1186/1471-2164-14-743 (2013). 24168272 PMC3870970

[b20] He Y. . Responses of the toll-like receptor and melanoma differentiation-associated protein 5 signaling pathways to avian infectious bronchitis virus infection in chicks. Virol. Sin. 31, 57–68 (2016). 26920710 10.1007/s12250-015-3696-y PMC7090632

[b21] Dar A. . Transcriptional analysis of avian embryonic tissues following infection with avian infectious bronchitis virus. Virus Res. 110, 41–55 (2005). 15845254 10.1016/j.virusres.2005.01.006 PMC7114260

[b22] Huang Y. P. & Wang C. H. Sequence changes of infectious bronchitis virus isolates in the 3′ 7.3 kb of the genome after attenuating passage in embryonated eggs. Avian Pathol. 36, 59–67 (2007). 17364511 10.1080/03079450601110015

[b23] Wang C. H., Hsieh M. C. & Chang P. C. Isolation, pathogenicity, and H120 protection efficacy of infectious bronchitis viruses isolated in Taiwan. Avian Dis. 40, 620–625 (1996). 8883793

[b24] Wang C. H. & Huang Y. C. Relationship between serotypes and genotypes based on the hypervariable region of the S1 gene of infectious bronchitis virus. Arch. Virol. 145, 291–300 (2000). 10752554 10.1007/s007050050024 PMC7086635

[b25] Kant A. . Location of antigenic sites defined by neutralizing monoclonal antibodies on the S1 avian infectious bronchitis virus glycopolypeptide. J. Gen. Virol. 73, 591–596 (1992). 1372036 10.1099/0022-1317-73-3-591

[b26] Cavanagh D. . Location of the amino acid differences in the S1 spike glycoprotein subunit of closely related serotypes of infectious bronchitis virus. Avian Pathol. 21, 33–43 (1992). 18670913 10.1080/03079459208418816

[b27] Cavanagh D., Elus M. M. & Cook J. K. Relationship between sequence variation in the S1 spike protein of infectious bronchitis virus and the extent of cross-protection *in vivo*. Avian Pathol. 26, 63–74 (1997). 18484262 10.1080/03079459708419194

[b28] Callison S. A., Jackwood M. W. & Hilt D. A. Infectious bronchitis virus S2 gene sequence variability may affect S1 subunit specific antibody binding. Virus Genes 19, 143–151 (1999). 10541018 10.1023/A:1008179208217 PMC7089226

[b29] Grosse B. & Siddell S. G. Single amino acid changes in the S2 subunit of the MHV surface glycoprotein confer resistance to neutralization by S1 subunit-specific monoclonal antibody. Virology 202, 814–824 (1994). 8030244 10.1006/viro.1994.1403

[b30] Casais R., Dove B., Cavanagh D. & Britton P. Recombinant avian infectious bronchitis virus expressing a heterologous spike gene demonstrates that the spike protein is a determinant of cell tropism. J. Virol. 77, 9084–9089 (2003). 12885925 10.1128/JVI.77.16.9084-9089.2003 PMC167237

[b31] Otsuki K., Nakamura T., Kubota N., Kawaoka Y. & Tsubokura M. Comparison of two strains of avian infectious bronchitis virus for their interferon induction, viral growth and development of virus-neutralizing antibody in experimentally-infected chickens. Vet. Microbiol. 15, 31–40 (1987). 2449761 10.1016/0378-1135(87)90126-x

[b32] Rose K. M., Elliott R., Martínez-Sobrido L., García-Sastre A. & Weiss S. R. Murine coronavirus delays expression of a subset of interferon-stimulated genes. J. Virol. 84, 5656–5669 (2010). 20357099 10.1128/JVI.00211-10 PMC2876584

[b33] Devaraj S. G. . Regulation of IRF-3-dependent innate immunity by the papain-like protease domain of the severe acute respiratory syndrome coronavirus. J. Biol. Chem. 282, 32208–32221 (2007). 17761676 10.1074/jbc.M704870200 PMC2756044

[b34] Versteeg G. A., Bredenbeek P. J., van den Worm S. H. & Spaan W. J. Group 2 coronaviruses prevent immediate early interferon induction by protection of viral RNA from host cell recognition. Virology 361, 18–26 (2007). 17316733 10.1016/j.virol.2007.01.020 PMC7103335

[b35] Kint J. . Activation of the chicken type I interferon response by infectious bronchitis coronavirus. J. Virol. 89, 1156–1167 (2015). 25378498 10.1128/JVI.02671-14 PMC4300645

[b36] Hodgson T., Britton P. & Cavanagh D. Neither the RNA nor the proteins of open reading frames 3a and 3b of the coronavirus infectious bronchitis virus are essential for replication. J. Virol. 80, 296–305 (2006). 16352554 10.1128/JVI.80.1.296-305.2006 PMC1317528

[b37] Kint J. . Infectious bronchitis coronavirus inhibits STAT1 signaling and requires accessory proteins for resistance to type I interferon activity. J. Virol. 89, 12047–12057 (2015). 26401035 10.1128/JVI.01057-15 PMC4645315

[b38] Kint J. . Infectious bronchitis coronavirus limits interferon production by inducing a host shutoff that requires accessory protein 5b. J. Virol. 90, 7519–7528 (2016). 27279618 10.1128/JVI.00627-16 PMC4984617

[b39] Han Z. . Altered pathogenicity of a tl/CH/LDT3/03 genotype infectious bronchitis coronavirus due to natural recombination in the 5′- 17kb region of the genome. Virus Res. 213, 140–148 (2016). 26616599 10.1016/j.virusres.2015.11.021 PMC7114521

[b40] Wang Y. . Coronavirus nsp10/nsp16 methyltransferase can be targeted by nsp10-derived peptide *in vitro* and *in vivo* to reduce replication and pathogenesis. J. Virol. 89, 8416–8427 (2015). 26041293 10.1128/JVI.00948-15 PMC4524257

[b41] Becares M. . Mutagenesis of coronavirus nsp14 reveals its potential role in modulation of the innate immune response. J. Virol. 90, 5399–5414 (2016). 27009949 10.1128/JVI.03259-15 PMC4934755

[b42] Kamitani W. . Severe acute respiratory syndrome coronavirus nsp1 protein suppresses host gene expression by promoting host mRNA degradation. Proc. Natl. Acad. Sci. USA 103, 12885–12890 (2006). 16912115 10.1073/pnas.0603144103 PMC1568942

[b43] Kameka A. M., Haddadi S., Kim D. S., Cork S. C. & Abdul-Careem M. F. Induction of innate immune response following infectious bronchitis corona virus infection in the respiratory tract of chickens. Virology 450-451, 114–121 (2014). 24503073 10.1016/j.virol.2013.12.001 PMC7111962

[b44] Menachery V. D. . Attenuation and restoration of severe acute respiratory syndrome coronavirus mutant lacking 2′-o-methyltransferase activity. J. Virol. 88, 4251–4264 (2014). 24478444 10.1128/JVI.03571-13 PMC3993736

[b45] Zalinger Z. B., Elliott R., Rose K. M. & Weiss S. R. MDA5 is critical to host defense during infection with murine coronavirus. J. Virol. 89, 12330–12340 (2015). 26423942 10.1128/JVI.01470-15 PMC4665247

[b46] Okabayashi T. . Cytokine regulation in SARS coronavirus infection compared to other respiratory virus infections. J. Med. Virol. 78, 417–424 (2006). 16482545 10.1002/jmv.20556 PMC7166776

[b47] Chen H. W., Huang Y. P. & Wang C. H. Identification of Taiwan and China-like recombinant avian infectious bronchitis viruses in Taiwan. Virus Res. 140, 121–129 (2009). 19100792 10.1016/j.virusres.2008.11.012 PMC7126714

[b48] Chen H. W. & Wang C. H. A multiplex reverse transcriptase-PCR assay for the genotyping of avian infectious bronchitis viruses. Avian Dis. 54, 104–108 (2010). 20408407 10.1637/8954-060609-Reg.1

[b49] Reed L. J. & Muench H. A simple method of estimating fifty per cent endpoints. Am. J. Epidemiol. 27, 493–497 (1938).

[b50] Tamura K., Dudley J., Nei M. & Kumar S. MEGA4: molecular evolutionary genetics analysis (MEGA) software version 4.0. Mol. Biol. Evol. 24, 1596–1599 (2007). 17488738 10.1093/molbev/msm092

[b51] Archetti I. & Horsfall F. L. Persistent antigenic variation of influenza A viruses after incomplete neutralization in ovo with heterologous immune serum. J. Exp. Med. 92, 441–462 (1950). 14778924 10.1084/jem.92.5.441 PMC2135986

[b52] Choi K. S. . Pathogenicity and antigenicity of a new variant of Korean nephropathogenic infectious bronchitis virus. J. Vet. Sci. 10, 357 (2009). 19934604 10.4142/jvs.2009.10.4.357 PMC2807275

[b53] Avellaneda G. E., Villegas P., Jackwood M. W. & King D. J. *In vivo* evaluation of the pathogenicity of field isolates of infectious bronchitis virus. Avian Dis. 38, 589–597 (1994). 7832713

[b54] Kapczynski D. R., Jiang H. J. & Kogut M. H. Characterization of cytokine expression induced by avian influenza virus infection with real-time RT-PCR. Methods Mol. Biol. 1161, 217–233 (2014). 24899432 10.1007/978-1-4939-0758-8_18

[b55] Livak K. J. & Schmittgen T. D. Analysis of relative gene expression data using real-time quantitative PCR and the 2 (T)(-Delta Delta C) method. Methods 25, 402–408 (2001). 11846609 10.1006/meth.2001.1262

