# Supplementary material for: Identification of an infectious bronchitis coronavirus strain exhibiting a classical genotype but altered antigenicity, pathogenicity, and innate immunity profile
Source: Sci Rep. 2016 Nov 23;6:37725. doi: 10.1038/srep37725 (PMC5120290; doi:10.1038/srep37725)
Supplement: supplementary Tables and Figures [file srep37725-s1.pdf]

Identification of an infectious bronchitis coronavirus strain exhibiting a classical genotype but altered antigenicity, pathogenicity, and innate immunity profile

Shu-Yi Lin[1], Yao-Tsun Li[1], You-Ting Chen[1], Ting-Chih Chen[1], Che-Ming J. Hu[2,3], and Hui-Wen Chen[1,3]*

[1]Department of Veterinary Medicine, National Taiwan University, Taipei, Taiwan
[2]Institute of Biomedical Sciences, Academia Sinica, Taipei, Taiwan
[3]Research Center for Nanotechnology and Infectious Diseases, Taipei, Taiwan

Supplementary Figure S1. Phylogenetic analyses of full length, E (envelope), M (membrane), and N (nucleocapsid) protein genes of IBV strains. The phylogenetic trees were constructed using the MEGA version 4 by the neighbor-joining method (bootstrapping for 1,000 replicates with its value >70%). The 3575/08 and 2575/98 strains analyzed in this study are indicated with red triangles. (AU: Australia, CN: China, NL: Netherlands, TW: Taiwan, UK: United Kingdom, US: United States, WA: Western Africa)

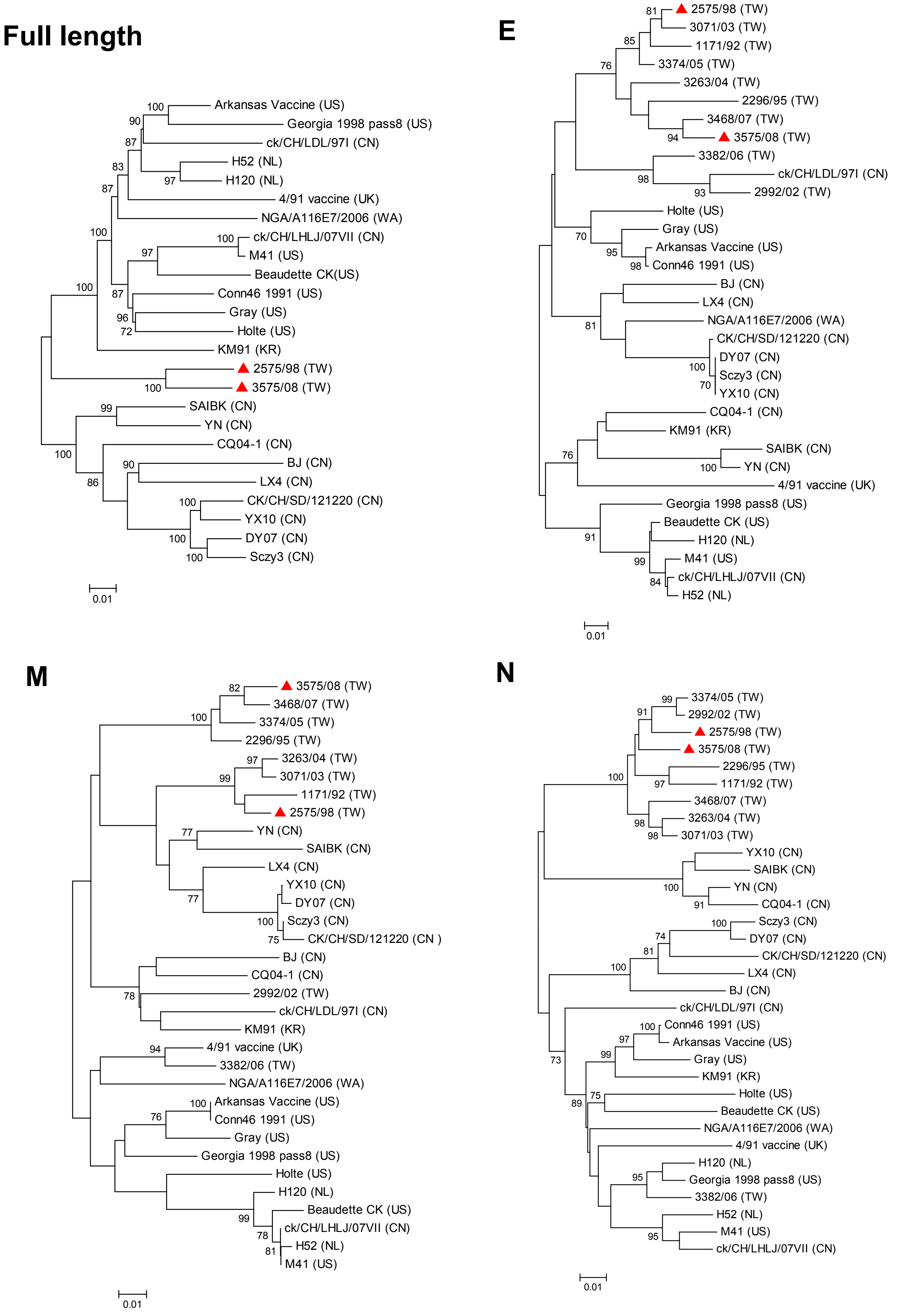

Supplementary Figure S2. Venn diagram of immune genes stimulated by IBV 3575/08, 2575 WT and 2575 AT at 24 hpi.

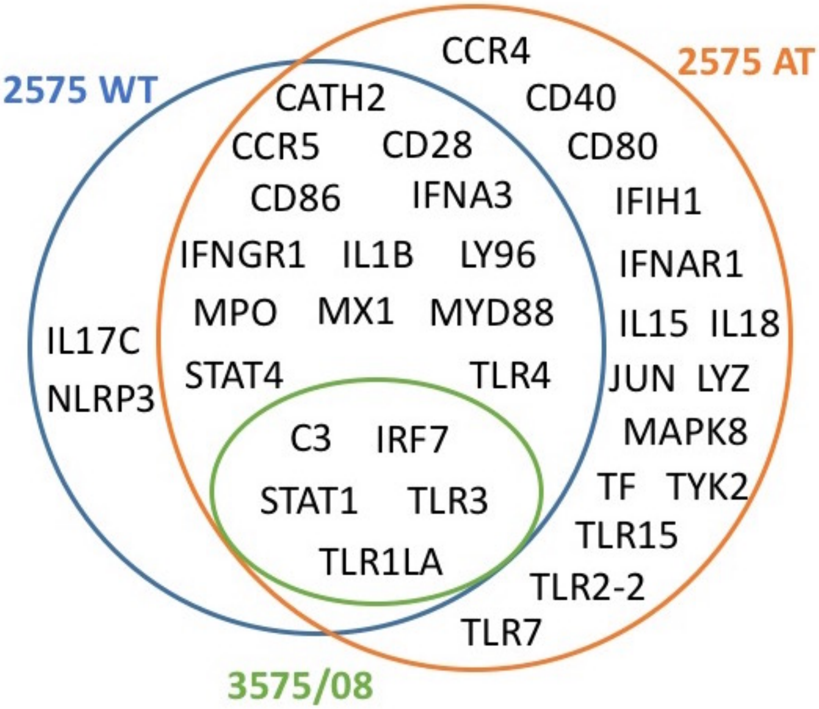

Supplementary Table S1. Cross neutralization test of IBVs. Homologous titers are indicated in bold.

IBV strain		Virus			
		3575/08 (TW-I)	2575/98 (TW-I)	2296/95 (TW-II)	H120 (Mass)
Serum	3575/08 (TW-I)	**8192**	102	34	16
	2575/98 (TW-I)	128	**1475**	108	16
	2296/95 (TW-II)	140	101	**512**	203
	H120 (Mass)	32	32	22	**531**

Supplementary Table S2. Quantitative PCR Array data from infected chicken lungs. A RT^2 Profiler Chicken Innate & Adaptive Immune Responses PCR Array (PAGG-052ZG; SABiosciences) was performed following the manufacturer's protocol. Complete list of 84 genes that were examined in the PCR array is presented. Fold change and its 95% confidence interval (CI) were calculated based upon naive chickens for IBV 3575/08, 2575 WT or 2575 AT-infected chickens using software provided by the manufacturer at 24h and 48h post infection. Numbers in red indicate fold change greater than 3.

Gene sym.		24 hpi						48 hpi					
		3575/08		2575 WT		2575 AT		3575/08		2575 WT		2575 AT	
	Description	Fold change	95% CI	Fold change	95% CI	Fold change	95% CI	Fold change	95% CI	Fold change	95% CI	Fold change	95% CI
C3	Complement component 3	5.00	(2.23, 7.76)	3.01	(2.23, 5.50)	8.12	(0.00001, 23.01)	8.02	(0.00001, 17.85)	0.99	(0.92, 1.05)	1.53	(0.00001, 3.43)
C5AR1	Complement component 5a receptor 1	1.08	(0.37, 1.79)	1.60	(0.07, 3.13)	1.92	(1.08, 2.76)	2.35	(0.38, 4.32)	1.73	(1.4, 2.08)	1.31	(0.93, 1.69)
CATH2	Cathelicidin antimicrobial peptide	2.07	(0.00001, 4.80)	6.10	(2.01, 10.19)	8.12	(0.00001, 22.12)	10.48	(0.18, 20.78)	8.75	(1.72, 20.25)	2.15	(0.57, 3.73)
CASP1	Caspase 1, apoptosis-related cysteine peptidase	0.95	(0.45, 1.45)	1.05	(0.26, 1.84)	1.83	(1.02, 2.64)	1.13	(0.11, 2.15)	0.88	(0.75, 1.01)	0.98	(0.65, 1.31)
CASP8	Caspase 8, apoptosis-related cysteine peptidase	0.99	(0.55, 1.43)	0.89	(0.49, 1.29)	1.45	(0.84, 2.06)	0.69	(0.35, 1.03)	0.61	(0.46, 0.77)	0.87	(0.53, 1.21)
CCL4	Chemokine (C-C motif) ligand 4	1.47	(0.44, 2.50)	2.67	(1.48, 3.86)	1.92	(1.08, 2.76)	2.68	(0.00001, 5.48)	1.73	(1.4, 2.08)	1.74	(0.62, 2.86)
CCL5	Chemokine (C-C motif) ligand 5	1.07	(0.36, 1.78)	1.87	(0.67, 3.07)	1.8	(0.91, 2.69)	2.20	(0.29, 4.11)	1.62	(1.31, 1.95)	1.23	(0.77, 1.69)
CCR4	Chemokine (C-C motif) receptor 4	1.10	(0.40, 1.80)	2.24	(1.20, 3.28)	3.34	(0.00001, 6.97)	2.33	(0.37, 4.29)	1.71	(1.39, 2.06)	1.30	(0.91, 1.69)
CCR5	Chemokine (C-C motif) receptor 5	2.74	(0.00001, 5.90)	4.77	(0.79, 8.75)	12.03	(0.00001, 37.15)	2.96	(0.00001, 7.80)	1.43	(1.16, 1.72)	1.49	(0.48, 2.50)
CCR6	Chemokine (C-C motif) receptor 6	1.08	(0.37, 1.79)	1.30	(0.00001, 3.03)	1.92	(1.08, 2.76)	2.35	(0.38, 4.32)	1.73	(1.4, 2.08)	1.31	(0.93, 1.69)
CCR8	Chemokine (C-C motif) receptor 8	0.89	(0.26, 1.52)	2.20	(1.07, 3.33)	1.92	(1.13, 2.71)	1.94	(0.24, 3.64)	1.73	(1.6, 1.85)	1.20	(0.71, 1.69)
CD14	CD14 molecule	1.08	(0.37, 1.79)	1.30	(0.00001, 3.03)	1.92	(1.08, 2.76)	2.35	(0.38, 4.32)	1.73	(1.4, 2.08)	1.31	(0.93, 1.69)
CD28	CD28 molecule	1.72	(0.00001, 3.48)	3.29	(1.19, 5.39)	14.47	(6.42, 22.52)	3.99	(0.00001, 11.75)	2.56	(1.86, 3.34)	3.47	(1.38, 5.56)
CD4	CD4 molecule	1.71	(0.19, 3.23)	2.84	(1.43, 4.25)	2.95	(1.26, 4.64)	2.66	(0.96, 4.36)	1.73	(1.4, 2.08)	1.77	(0.70, 2.84)
CD40	CD40 molecule, TNF receptor superfamily member 5	1.65	(0.00001, 3.92)	2.92	(0.00001, 6.11)	4.45	(1.28, 7.62)	3.84	(0.00001, 10.30)	1.35	(1.09, 1.63)	1.78	(0.17, 3.39)
CD40LG	CD40 ligand (TNF superfamily, member 5, hyper-IgM syndrome)	1.07	(0.24, 1.90)	2.97	(0.33, 5.61)	1.9	(0.51, 3.29)	2.37	(0.00001, 5.29)	1.48	(1.16, 1.83)	1.51	(0.32, 2.70)
CD80	CD80 molecule	1.01	(0.46, 1.56)	2.42	(1.47, 3.37)	3.05	(0.92, 5.18)	2.47	(0.76, 4.18)	1.49	(1.21, 1.79)	1.20	(1.02, 1.38)
CD86	CD86 molecule	1.89	(0.12, 3.66)	4.10	(0.38, 7.82)	8.25	(2.90, 13.60)	2.77	(0.00001, 5.81)	1.73	(1.4, 2.08)	1.73	(0.62, 2.84)
CD8A	CD8a molecule	0.71	(0.22, 1.20)	2.24	(0.45, 4.03)	1.56	(0.92, 2.20)	1.54	(0.20, 2.88)	1.13	(0.92, 1.36)	0.86	(0.54, 1.18)
CRP	C-reactive protein, pentraxin-related	1.08	(0.37, 1.79)	1.30	(0.00001, 3.03)	1.92	(1.08, 2.76)	2.35	(0.38, 4.32)	1.73	(1.4, 2.08)	1.31	(0.93, 1.69)
CSF2	Granulocyte-macrophage colony-stimulating factor	1.08	(0.37, 1.79)	1.30	(0.00001, 3.03)	1.92	(1.08, 2.76)	2.35	(0.38, 4.32)	1.73	(1.4, 2.08)	1.31	(0.93, 1.69)
CXCL12	Chemokine (C-X-C motif) ligand 12 (stromal cell-derived factor 1)	0.87	(0.00001, 1.85)	0.56	(0.00001, 1.35)	1.43	(0.50, 2.36)	1.98	(0.00001, 4.37)	1.53	(1.43, 1.63)	0.68	(0.23, 1.13)
CXCR4	Chemokine (C-X-C motif) receptor 4	0.79	(0.05, 1.53)	1.14	(0.19, 2.09)	0.77	(0.00001, 1.93)	0.95	(0.50, 1.40)	0.58	(0.55, 0.6)	0.48	(0.03, 0.93)
FAS	Fas (TNF receptor superfamily, member 6)	1.47	(0.00001, 2.97)	1.85	(0.10, 3.60)	2.35	(0.01, 4.69)	2.67	(0.00001, 6.34)	1.85	(1.15, 2.69)	0.77	(0.13, 1.41)
FASLG	Fas ligand (TNF superfamily, member 6)	1.08	(0.37, 1.79)	2.02	(0.84, 3.20)	1.97	(1.18, 2.76)	4.17	(1.77, 6.57)	1.73	(1.4, 2.08)	1.31	(0.93, 1.69)
GATA3	GATA binding protein 3	1.40	(0.09, 2.71)	2.29	(0.29, 4.29)	1.39	(0.33, 2.45)	1.04	(0.00001, 2.30)	1.01	(0.77, 1.28)	0.78	(0.03, 1.53)
IFIH1	Interferon induced with helicase C domain 1	1.97	(0.00001, 4.34)	1.88	(0.92, 2.84)	3.35	(1.92, 4.78)	1.20	(0.25, 2.15)	1.60	(1.38, 1.83)	0.75	(0.34, 1.16)
IFNA3	Interferon	1.20	(0.33, 2.08)	3.09	(2.04, 4.15)	27.63	(9.59, 45.67)	5.62	(1.39, 9.85)	1.36	(1.1, 1.64)	14.66	(1.99, 27.33)
IFNAR1	Interferon (alpha, beta and omega) receptor 1	1.10	(0.00001, 2.34)	2.58	(0.00001, 5.25)	3.59	(0.81, 6.37)	2.39	(0.02, 4.76)	1.90	(1.28, 2.62)	0.95	(0.00001, 1.96)
IFNB	Interferon beta	1.21	(0.47, 1.95)	2.17	(1.09, 3.25)	1.92	(1.08, 2.76)	2.35	(0.38, 4.32)	1.73	(1.4, 2.08)	1.31	(0.93, 1.69)
IFNG	Interferon, gamma	1.08	(0.37, 1.79)	1.30	(0.00001, 3.03)	1.92	(1.08, 2.76)	2.35	(0.38, 4.32)	1.73	(1.4, 2.08)	1.31	(0.93, 1.69)
IFNGR1	Interferon gamma receptor 1	1.31	(0.00001, 3.55)	3.54	(0.00001, 7.40)	3.67	(1.96, 5.38)	3.36	(0.00001, 7.12)	1.61	(0.79, 2.71)	1.88	(0.43, 3.33)
IL10	Interleukin 10	1.08	(0.37, 1.79)	1.30	(0.00001, 3.03)	1.92	(1.08, 2.76)	2.35	(0.38, 4.32)	1.73	(1.4, 2.08)	1.31	(0.93, 1.69)
IL13	Interleukin 13	0.13	(0.00001, 0.70)	0.17	(0.00001, 0.94)	0.22	(0.00001, 1.18)	0.34	(0.00001, 1.83)	0.43	(0.2, 0.43)	0.15	(0.00001, 0.81)
IL15	Interleukin 15	1.45	(0.00001, 2.98)	2.02	(0.44, 3.60)	5.06	(4.58, 5.54)	2.75	(0.14, 5.36)	1.29	(0.87, 3.38)	1.34	(0.98, 1.70)
IL18	Interleukin 18 (interferon-gamma-inducing factor)	1.46	(0.44, 2.48)	2.12	(0.68, 3.56)	3.21	(2.16, 4.26)	2.20	(0.93, 3.47)	1.75	(1.66, 1.84)	1.27	(0.88, 1.66)
IL1B	Interleukin 1, beta	1.44	(0.25, 2.63)	3.83	(0.00001, 7.66)	4.96	(0.00001, 9.99)	2.79	(1.26, 4.32)	1.59	(1.29, 1.91)	1.53	(0.55, 2.51)
IL1R1	Interleukin 1 receptor, type I	0.54	(0.00001, 1.10)	1.18	(0.35, 2.01)	1.7	(0.00001, 3.54)	1.07	(0.00, 2.14)	0.58	(0.57, 0.59)	1.00	(0.28, 1.72)
IL2	Interleukin 2	0.95	(0.39, 1.51)	1.59	(0.49, 2.69)	1.69	(1.13, 2.25)	2.07	(0.44, 3.70)	1.52	(1.23, 1.83)	1.15	(1.08, 1.22)
IL4	Interleukin 4	0.99	(0.39, 1.59)	1.58	(0.34, 2.82)	1.75	(1.14, 2.36)	2.15	(0.44, 3.86)	1.58	(1.28, 1.9)	1.20	(1.06, 1.34)
IL5	Interleukin 5	1.08	(0.37, 1.79)	1.30	(0.00001, 3.03)	1.92	(1.08, 2.76)	2.35	(0.38, 4.32)	1.73	(1.4, 2.08)	1.32	(0.94, 1.70)
IL6	Interleukin 6 (interferon, beta 2)	1.08	(0.37, 1.79)	2.64	(0.00001, 7.59)	1.92	(1.08, 2.76)	2.35	(0.38, 4.32)	1.73	(1.4, 2.08)	1.31	(0.93, 1.69)
IL8L1	Interleukin 8	0.95	(0.23, 1.67)	1.14	(0.00001, 2.71)	1.68	(0.72, 2.64)	2.06	(0.17, 3.95)	1.52	(1.23, 1.82)	1.15	(0.61, 1.69)
IRAK4	Interleukin-1 receptor-associated kinase 4	1.00	(0.10, 1.90)	1.09	(0.46, 1.72)	1.97	(1.13, 2.81)	0.67	(0.00001, 1.51)	0.66	(0.39, 1.01)	0.51	(0.00001, 1.31)
IRF1	Interferon regulatory factor 1	2.26	(0.14, 4.38)	2.61	(0.97, 4.25)	1.86	(0.55, 3.17)	1.05	(0.00001, 2.45)	1.64	(1.3, 2.01)	0.45	(0.00001, 0.98)
IRF6	Interferon regulatory factor 6	1.23	(0.31, 2.15)	0.71	(0.13, 1.29)	1.55	(0.52, 2.58)	1.18	(0.00001, 2.48)	1.49	(1.37, 1.6)	1.33	(0.81, 1.85)
IRF7	Interferon regulatory factor 7	3.44	(0.00001, 8.01)	11.26	(0.40, 22.12)	4.9	(0.53, 9.27)	2.18	(0.00001, 4.63)	1.71	(1.28, 2.18)	1.40	(0.00001, 3.19)
ITGB2	Integrin, beta 2 (complement component 3 receptor 3 and 4 subunit)	1.08	(0.37, 1.79)	1.30	(0.00001, 3.03)	1.92	(1.08, 2.76)	3.00	(1.55, 4.45)	1.73	(1.4, 2.08)	1.31	(0.93, 1.69)
JAK2	Janus kinase 2 (a protein tyrosine kinase)	0.88	(0.37, 1.39)	1.59	(0.70, 2.48)	1.43	(0.66, 2.20)	1.31	(0.00001, 3.04)	0.46	(0.44, 0.49)	0.68	(0.18, 1.18)
JUN	Jun proto-oncogene	1.16	(0.00001, 2.79)	1.77	(0.35, 3.19)	3.8	(0.00001, 7.81)	1.93	(0.27, 3.59)	0.50	(0.39, 0.63)	2.18	(0.95, 3.41)
LITAF	Lipopolysaccharide-induced TNF factor	0.54	(0.19, 0.89)	1.08	(0.22, 1.94)	1.11	(0.50, 1.72)	0.81	(0.11, 1.51)	1.01	(0.69, 1.38)	0.26	(0.16, 0.36)
IL17C	Interleukin-17C-like	1.08	(0.37, 1.79)	4.56	(0.00001, 18.66)	1.92	(1.08, 2.76)	2.35	(0.38, 4.32)	1.73	(1.4, 2.08)	1.31	(0.93, 1.69)
STAT6	Signal transducer and transcription activator 6-like	1.73	(0.00001, 3.51)	2.58	(0.40, 4.76)	2.04	(0.00001, 4.12)	2.27	(0.10, 4.44)	1.34	(0.74, 2.1)	1.05	(0.00001, 2.21)
TYK2	Similar to MGC83617 protein	1.44	(0.00001, 4.04)	2.61	(0.00001, 6.44)	3.5	(0.00001, 7.61)	1.82	(0.00001, 4.79)	0.44	(0.36, 0.53)	1.96	(0.00001, 4.34)
TF	Lactotransferrin	1.14	(0.00001, 2.91)	1.92	(0.87, 2.97)	3.01	(0.46, 5.56)	4.67	(1.83, 7.51)	1.22	(0.78, 1.75)	1.75	(0.65, 2.85)
LY96	Lymphocyte antigen 96	2.36	(0.00001, 5.18)	3.49	(0.00001, 7.92)	12.83	(3.23, 22.43)	2.54	(0.00001, 6.14)	2.52	(1.2, 4.31)	1.59	(0.00001, 3.20)
LYZ	Lysozyme (renal amyloidosis)	1.82	(0.00001, 4.93)	2.43	(0.00001, 6.15)	4.14	(0.00001, 10.79)	3.03	(0.00001, 7.04)	1.99	(0.00001, 6.46)	1.44	(0.00001, 3.30)
MAPK1	Mitogen-activated protein kinase 1	1.47	(0.80, 2.14)	1.23	(0.55, 1.91)	0.99	(0.57, 1.41)	1.25	(0.00001, 2.66)	1.16	(0.92, 1.42)	0.77	(0.33, 1.21)
MAPK14	Mitogen-activated protein kinase 14	1.78	(0.50, 3.06)	2.11	(1.11, 3.11)	2.79	(1.59, 3.99)	2.00	(0.09, 3.91)	1.14	(0.94, 1.35)	1.06	(0.53, 1.59)
MAPK8	Mitogen-activated protein kinase 8	2.71	(0.00001, 7.20)	2.40	(0.00001, 6.82)	8.52	(0.00001, 20.87)	3.04	(0.00001, 9.18)	0.79	(0.52, 1.11)	3.28	(0.00001, 8.05)
MBL2	Mannose-binding lectin (protein C) 2, soluble	1.08	(0.37, 1.79)	1.30	(0.00001, 3.03)	1.92	(1.08, 2.76)	4.56	(2.41, 6.71)	1.97	(1.92, 2)	1.33	(0.94, 1.72)
MPO	Myeloperoxidase	1.13	(0.42, 1.84)	3.10	(1.22, 4.98)	6.52	(3.32, 9.72)	2.35	(0.38, 4.32)	1.87	(1.34, 2.47)	1.63	(0.87, 2.39)
MX1	Myxovirus resistance 1, interferon-inducible protein p78 (mouse)	2.75	(0.28, 5.22)	6.54	(4.28, 8.80)	8.56	(4.09, 13.03)	2.16	(0.00001, 5.31)	2.16	(0.13, 5.83)	0.31	(0.00001, 0.83)
MYD88	Myeloid differentiation primary response gene (88)	1.98	(0.00001, 4.00)	4.58	(0.38, 8.78)	3.61	(0.27, 6.95)	3.72	(0.00001, 8.31)	0.67	(0.57, 0.78)	1.82	(0.00001, 4.00)
NFKB1	Nuclear factor of kappa light polypeptide gene enhancer in B-cells 1	0.84	(0.23, 1.45)	0.88	(0.02, 1.74)	1.76	(0.51, 3.01)	1.05	(0.00001, 2.18)	0.96	(0.7, 1.25)	0.78	(0.31, 1.25)
NFKBIA	Nuclear factor of kappa light polypeptide gene enhancer in B-cells inhibitor, alpha	0.46	(0.15, 0.77)	0.68	(0.30, 1.06)	0.76	(0.17, 1.35)	0.67	(0.14, 1.20)	0.55	(0.55, 0.56)	0.31	(0.10, 0.52)
NLRP3	NLR family, pyrin domain containing 3	2.86	(0.51, 5.21)	3.66	(2.05, 5.27)	2.73	(1.37, 4.09)	2.40	(0.37, 4.43)	1.73	(1.4, 2.08)	1.37	(0.95, 1.79)
NOD1	Nucleotide-binding oligomerization domain containing 1	1.50	(0.12, 2.88)	1.35	(0.11, 2.59)	2.29	(0.08, 4.50)	1.17	(0.00001, 2.87)	0.81	(0.47, 1.23)	0.54	(0.00001, 1.18)
PTGS2	Prostaglandin-endoperoxide synthase 2	0.79	(0.16, 1.42)	1.39	(0.51, 2.27)	1.15	(0.33, 1.97)	1.49	(0.48, 2.50)	0.88	(0.71, 1.06)	0.67	(0.32, 1.02)
RAG1	Recombination activating gene 1	1.00	(0.28, 1.72)	1.16	(0.00001, 2.75)	1.89	(0.99, 2.79)	2.21	(0.04, 4.38)	1.54	(1.24, 1.85)	1.21	(0.67, 1.75)
SLC11A1	Solute carrier family 11, member 1	1.37	(0.49, 2.25)	2.56	(1.41, 3.71)	2.33	(1.58, 3.08)	2.35	(0.38, 4.32)	1.73	(1.4, 2.08)	1.68	(0.69, 2.67)
STAT1	Signal transducer and activator of transcription 1, 91kDa	3.28	(0.00001, 6.96)	6.91	(1.15, 12.67)	5.6	(1.50, 9.70)	2.16	(0.00001, 4.79)	2.67	(0.95, 5.2)	0.95	(0.02, 1.88)
STAT3	Signal transducer and activator of transcription 3	0.81	(0.43, 1.19)	1.61	(0.58, 2.64)	0.93	(0.20, 1.66)	1.53	(0.00001, 3.26)	0.97	(0.83, 1.11)	0.44	(0.02, 0.86)
STAT4	Signal transducer and activator of transcription 4	1.54	(0.10, 2.98)	4.20	(0.58, 7.82)	3.21	(0.97, 5.45)	2.37	(0.00001, 4.85)	1.57	(1.27, 1.88)	1.19	(0.69, 1.69)
TICAM1	Toll-like receptor adaptor molecule 1	1.38	(0.63, 2.13)	1.35	(0.24, 2.46)	2.89	(2.08, 3.70)	1.20	(0.00001, 2.59)	1.33	(0.68, 2.17)	1.60	(0.71, 2.49)
TLR15	Toll-like receptor 15	0.86	(0.11, 1.61)	1.03	(0.00001, 2.52)	3.68	(0.05, 7.31)	1.92	(0.05, 3.79)	1.37	(1.11, 1.65)	1.04	(0.37, 1.71)
TLR21	Toll-like receptor 21	1.08	(0.37, 1.79)	1.41	(0.00001, 3.08)	1.92	(1.08, 2.76)	2.35	(0.38, 4.32)	1.73	(1.4, 2.08)	1.31	(0.93, 1.69)
TLR2-2	Toll-like receptor 2	1.39	(0.02, 2.76)	2.16	(0.26, 4.06)	7.84	(2.97, 12.71)	1.82	(0.14, 3.50)	1.34	(1.08, 1.61)	1.46	(0.17, 2.75)
TLR3	Toll-like receptor 3	4.52	(0.00001, 10.52)	5.58	(0.00001, 11.42)	12.19	(7.62, 16.76)	2.37	(0.13, 4.61)	6.03	(4.65, 7.55)	1.64	(1.07, 2.21)
TLR4	Toll-like receptor 4	1.51	(0.40, 2.62)	5.65	(0.71, 10.59)	5.88	(0.00001, 12.89)	3.78	(1.22, 6.34)	2.09	(1.95, 2.21)	2.39	(1.22, 3.56)
TLR5	Toll-like receptor 5	0.57	(0.00001, 1.27)	0.62	(0.00001, 1.98)	0.86	(0.00001, 1.99)	0.80	(0.00001, 2.08)	0.71	(0.43, 1.05)	0.29	(0.00001, 0.64)
TLR1LA	Toll-like receptor 1-like A	7.18	(4.02, 10.35)	3.84	(0.00001, 8.80)	18.52	(7.55, 29.49)	8.22	(2.68, 13.76)	4.47	(3.24, 5.87)	3.57	(0.67, 6.47)
TLR7	Toll-like receptor 7	0.95	(0.11, 1.79)	2.90	(0.16, 5.64)	7.99	(0.04, 15.94)	2.87	(0.76, 4.98)	1.79	(0.84, 3.07)	1.44	(0.15, 2.73)
TRAF6	TNF receptor-associated factor 6	2.17	(0.84, 3.50)	2.32	(0.19, 4.45)	1.83	(0.37, 3.29)	3.11	(0.00001, 7.14)	1.87	(1.51, 2.25)	1.02	(0.00001, 2.20)

Supplementary Table S3. Primers used to amplify the complete genomic sequence of IBV 3575/08.

Location[a]	Length (bp)	Forward primer (5'→3')	Reverse primer (5'→3')
1-3009	3009	ACTGAAAATAGATATTAATATATATC	CTTGCCGGGTCTTGTATAAG
2502-4403	1902	CACCAATGTCACAACTTGGT	CACGCCACTCAAGTATAACA
2985-5732	2748	TGGCTCTTATACAAGACCCG	AATGTCCACGTGTACTTCCA
5011-7691	2681	GAATGAAAGTTCTTTACCTG	TGGCTGTTACAATTGGACAG
7084-9024	1941	AGTGGAATATACAGGTGACG	CAAAGCCCATTAAGATTGTTG
8509-9961	1453	AATGCATTGCTGGCTTGTGT	CCTAAGTGGCACTGCCGT
9663-11669	2007	CACCATTTTCTACTAGTACTGC	CTACACCTGCTACGCAAGC
11572-15221	3650	CTTGACTAGGAATGGGCATAAT	ACTACACAGACGCCACAAG
14717-16297	1581	CCAAACAAGGTCTTGTAGCAG	CTTCTGGCAAGGCATTAATAGTA
16184-17725	1542	ACGATTGCACTCGTATAGTA	CATCCATTAAAAGTGGGTAGT
17505-20183	2679	GTTAGCGGACAATTTATGCA	GAAGCATTAACTGCTGTACA
20001-21037	1037	CGTAGTAGCCAATAATGGCA	GGATTTAGGCCGTCCTGATTT
20375-22875	2501	AAACTGAACAAAAGACAGACTTAG	CCTTCCTGCATATGACCAA
21969-23915	1947	TTCTGACTTCTCGTAATGA	TGTATTGTTCAGTTACCACA
23189-25686	2498	TTTTGTGGTAGTGGAAGACA	TTGAGTTAATAGTAGGGATTT
24532-26607	2076	GTTATTGTTAACGAGTTTCCWA	GCTGCACGAGCAATAAGA
25884-27462	1579	GGATTAGATTGTGTTTACTT	AGCTACATGCCTATCTTCC

[a]Locations are listed according to IBV strain 2575/98 (accession no. DQ646405).

Supplementary Table S4. IBV reference strains included in this study.

Strain	Accession no.	Country	Strain	Accession no.	Country
Vic	DQ490221[a]	Australia	1171/92	DQ646406[b]	Taiwan
Armidale	DQ490205[a]	Australia	2296/95	DQ646404[b]	Taiwan
Sczy3	JF732903	China	2575/98	DQ646405	Taiwan
YN	JF893452	China	2992/02	EU822340[b]	Taiwan
LX4	AY338732	China	3071/03	EU822340[b]	Taiwan
BJ	AY319651	China	3263/04	EU822338[b]	Taiwan
YX10	JX840411	China	3374/05	EU822338[b]	Taiwan
DY07	HM245923	China	3382/06	GQ229232[b]	Taiwan
CK/CH/SD/121220	KJ128295	China	3468/07	EU822338[b]	Taiwan
CQ04-1	HM245924	China	4/91 vaccine	KF377577	UK
SAIBK	DQ288927	China	Conn46 1991	FJ904719	USA
CK/CH/LHLJ/07VII	JF274479	China	Gray	GU393334	USA
GX-NN09032	JX897900	China	Holte	GU393336	USA
CK/CH/LDL/97I	JX195177	China	M41	DQ834384	USA
H52	EU817497	Netherlands	Beaudette CK	AJ311317	USA
H120	FJ888351	Netherlands	Georgia 1998 pass8	GQ504722	USA
KM91	JQ977698	South Korea	Arkansas Vaccine	GQ504721	USA
NGA/A116E7/2006	FN430415	Nigeria	--	--	--

[a]Sequences used for S gene comparison.
[b]Sequences used for S-N gene comparison.
